# Implications of water insecurity for food practices and health in contexts of migration and displacement: evidence from the US-Mexico borderlands

**DOI:** 10.3389/fnut.2026.1771924

**Published:** 2026-07-30

**Authors:** Megan A. Carney, Caylyn Rich, Deyanira Ibarra, Amber Wutich, Patrick Thomson

**Affiliations:** 1 University of Arizona, Tucson, AZ, United States; 2 Arizona State University, Tempe, AZ, United States

**Keywords:** community-engaged methodologies, displaced/migrant populations, food insecurity, resource insecurity, US-Mexico borderlands, water insecurity

## Abstract

**Introduction:**

This article examines the interconnected burdens of food and water insecurity among migrant and displaced populations in North America, highlighting critical gaps in current research and policy. While extensive scholarship demonstrates that these populations disproportionately experience food insecurity—defined as limited access to nutritionally sufficient and culturally acceptable food—water insecurity, or unreliable access to safe water for consumption and household needs, as experienced by these populations has received comparatively less attention. Emerging evidence shows that water insecurity is increasingly prevalent in migrant-receiving regions of the global north and frequently overlaps with food insecurity, particularly among low- income communities.

**Methods:**

Through community-based collaborative research with transborder, migrant, and refugee populations in the borderlands region of southern Arizona, we co-coordinated *pláticas*—a Chicana/Latina feminist methodological approach of gathering to build conversation that validates experiential knowledge—with community partners to assess experiences of and perspectives on water insecurity.

**Results:**

Although the research activities were designed primarily to assess experiences of and perspectives on water insecurity, our findings underscore the need to consider food and water insecurity together in migratory and transborder settings to more fully understand barriers to household and community wellbeing.

**Discussion:**

The article concludes by calling for a coordinated research and policy agenda that addresses these mutually reinforcing resource insecurities and develops more effective, multi—scalar interventions to support the health and livelihoods of those in migratory contexts, particularly in the global north.

## 1 Introduction

Dietary transformations and their health implications for migrant populations are often undergirded by uneven or unreliable access to food resources. A robust body of research firmly establishes how migrant populations are disproportionately burdened by food insecurity (1–3). Food insecurity, or lack of food that is nutritionally sufficient and culturally acceptable, has been shown to both inform decision-making around and punctuate experiences of migration, including among migrant-receiving settings in the global north characterized by high levels of social inequality. Whereas migration often serves as a coping mechanism for mitigating food insecurity, policy debates on food insecurity and migration are frequently bifurcated, “as if no one eats anything in the world of international migration, and no one moves anywhere in the world of food security” (4) (p. 342). Despite many people migrating as a response to food insecurity (5), and perceptions that experiences of food insecurity are uncommon in the global north, case studies highlight continued struggles with food security for migrants after resettlement in so-called host settings (1, 3, 6, 7).

By contrast, water insecurity only recently gained attention in studies of south-north migration (8–10), reflecting emerging evidence that water insecurity is increasingly prevalent in the global north and often emerges alongside food insecurity, particularly among low-income households. Water insecurity, or lack of access to safe and reliable sources of water for human consumption, compounds with and exacerbates conditions of food insecurity (11–15). Yet there is remarkably limited research on the compounding effects of food and water insecurity in migratory contexts.

Drawing on evidence from community-based collaborative research in the US-Mexico borderlands region of southern Arizona, this study examines community-level experiences of household water insecurity among migrant, transborder, and refugee communities with the aim of informing a more integrated research and policy agenda that addresses the compounding effects of food and water insecurity on migrant health and livelihoods in the global north. While the research activities described in this paper did not set out to center these considerations in seeking to understand local experiences with and perspectives on household- and community-level water insecurity, our findings suggest that a comprehensive approach is necessary for developing more effective policy solutions at various scales, advancing efforts to alleviate both food and water insecurity, and supporting migrant health and livelihoods more broadly.

## 2 Water insecurity among migrant populations in the global north

While the role of water insecurity in motivating and shaping migration patterns is understudied, migrants continue to face water insecurity in the global north (9, 16, 17). Despite the common assumption that water in the global north is “modern,” namely, that all water is clean, affordable, and universally accessible, in reality, water quality is often unreliable and governance inconsistent (18). Water quality violations are most prevalent in the US Southwest (19), as evidenced by the irregular or absent water quality monitoring on the US-Mexico border (20).

There is a clear correlation between housing, poverty, and water insecurity. In the US-Mexico borderlands for example, poverty poses a direct risk for incomplete plumbing and lack of access to piped running water, reinforcing conditions of plumbing poverty (21). Households of color are disproportionately affected, largely due to racialized policies that perpetuate unaffordable urban housing (22). Consequently, migrants with precarious citizenship who face differential access to resources through housing, linguistic, and economic discrimination are more likely to have incomplete plumbing, thus highlighting the link between citizenship or formal belonging and water security (18, 21, 23). Indeed, mixed-status households have higher rates of water insecurity than the general population (24).

These factors contribute to increased distrust in water safety and quality, and greater reliance on bottled water over tap water (25–28). Yet bottled water, and harvested rainwater, constitute “replacement” water, which is generally more expensive than tap water (18). Households with higher incomes in water-scarce communities in low- and middle-income countries can utilize certain social networks, such as free water through employment opportunities, or bartering with high-value assets to obtain water, and are better able to mitigate effects of water insecurity without spending more money (16). While the role of social networks has not been explicitly explored for migrants in water-insecure settings in the global north, it is likely that low-income and migrant communities shoulder greater burdens of increasing water costs (29, 30).

Altering hydration practices and relying on various coping strategies to meet water needs in situations of water insecurity may imply a variety of negative physiological and psychological health outcomes (31, 32). For example, collecting water diverts time from other domestic chores, as well as economic and social activities (9). Water system intermittency occurring at indeterminate and unpredictable times has psychosocial impacts (33). It portends negatively for health and hygiene when water users expect to have running water at certain times of the day to perform daily tasks but do not, either due to unreliable systems or intentional shut-offs (18). Perceptions of water safety and quality is understood through a plethora of contextual factors, and distrust in water systems is a reasonable response to situations where water security is lacking (18, 34, 35).

Previous studies have looked at short-term migration and long-term resettlement as responses to household food and water insecurity in the global south (36). However, few studies have explored the persistence of food and water insecurity as they affect migrant and displaced populations in the global north. Further research is useful for theory-building in the relatively young field of water insecurity in the global north and for developing applied interventions tailored to the needs of migratory and transborder communities.

### 2.1 The food-water insecurity nexus

Emerging efforts to evaluate water and food insecurity together indicate association through multiple pathways (11, 16). These include impacts from the lack of water for growing food, cooking and preparing food according to how much water is available, and high prices for food and water (37, 38). Allocating household incomes toward water impacts food insecurity by siphoning resources from food budgets and thereby elevating stress (16). Water insecurity may thus lead to reduced dietary intake either due to spending money on other needs like medicine or because water is not available to prepare or cook food (38). At the household level, people may experience piped water skepticism (39), or well-founded concerns about tap water quality and safety. Avoiding tap water, then, may lead to nutritional consequences by drinking other less healthy beverages in the same way that conditions of food insecurity may lead to people eating what is available rather than what is nutritious or desired (40). Evidence from research with unhoused populations suggests that marginalized communities may respond to water and food insecurity in unconventional ways, and subsequently face increased health risks related to bathing, drinking, and eating from contaminated sources (41–43). The effects of food and water insecurity intensify unpredictability and uncertainty which may lead to emotional or psychosocial stress (44–47).

## 3 Methodology

The Sonoran Desert spanning southern Arizona and northern Mexico faces high rates of evapotranspiration, prolonged periods of drought, and extreme summer temperatures. Combined with increasingly less rainfall and higher average temperatures, both surface and groundwater resources in southern Arizona are depleting more quickly than in the past (48). As the hottest and driest portion of the state, southern Arizona receives the majority of its annual precipitation during the North American Monsoon season, typically from July through September. July and August in particular account for up to half of total annual precipitation levels, leading to increased flooding which poses concerns for water quality from private wells during the monsoon season (48). Many rural areas in southern Arizona rely heavily on private, domestic wells for water access, which may tap shallow or declining aquifers sensitive to drought. In contrast, in urban settings such as Tucson, where the average annual precipitation is 10.1 inches (49), water is generally provided through municipal or investor-owned water utilities. Public water systems, defined as those with > 14 connections, are subject to financial regulation and quality standards. However, water quality is only regulated for factors that are known to affect health; as a result, utilities can provide water that—while safe to drink—is unpalatable.

A significant share of Arizona’s water supply is delivered through the Central Arizona Project (CAP) that transports water from the Colorado River hundreds of miles across the state. Statewide, approximately 41% of Arizona’s water supply comes from groundwater and 36% from the Colorado River, while the agricultural sector accounts for roughly 72% of total water use (50). The greater Tucson region is also supplied by local aquifers, such as the Santa Cruz River watershed, which has undergone long-term depletion due to historic over-pumping and limited natural recharge.

These complex and overlapping climatic and infrastructural conditions amplify challenges for water-insecure communities in the state. Despite innovative strategies to respond to these circumstances, indicators of poverty, energy and food security, childhood wellbeing and health continue to rank low in the US Southwest (51). Among the borderland counties of southern Arizona, including Pima, Cochise, Santa Cruz, and Yuma, 41% of the population identifies as Hispanic or Latino (52) and the average age is 39 years old (53). In 2023, the average poverty rate across these counties was 16.65% (53) compared to 11.7% for the state of Arizona (52).

Like much of the US-Mexico borderlands, southern Arizona is heavily militarized with border surveillance technologies and widespread presence of immigration enforcement constituting the norm. Border checkpoints are common, with arbitrary immigration checks allowed within a hundred miles of the border, and stops also reported beyond the hundred-mile limit; such surveillance and securitization measures contribute to a climate of heightened fear and anxiety and compound with other insecurities for mixed-status households and communities in the region (54). Despite nativist and populist discourse purporting that migrants hoard or “steal” resources from citizens, fears about the possibility of apprehension by immigration enforcement, detention, and deportation prevent many noncitizens from accessing essential services, such as health care or food assistance (55). Spaces of policing occur not only at the border, but also before arrival through a process of outsourcing, or pressuring transit countries including Mexico to enforce barriers to migration, as well as insourcing by maintaining liminal legal status for migrants after arrival (56).

The environmental and sociopolitical dynamics outlined above are important for understanding the broader context that informed our approach to community-based collaborative research to understanding lived experiences of water insecurity in the region, including deferring to the suggestion by community partners to employ *pláticas* as a primary method of data collection.

### 3.1 Pláticas

Community-based research emphasizes a process of identifying values among all partners at the beginning of the research process (57). Pláticas are “informal conversations that take place in one-on-one or group spaces” (58) (p. 117) and focus on the transfer of knowledge tied to meaning-making (59).

Grounded in feminist Chicana/Latina epistemologies, pláticas can be conceptualized as both methodology and method (60). As a methodology, the pláticas represent a stance that challenges dominant epistemological frameworks which position knowledge production as the exclusive terrain of academic experts (61). Pláticas recognize that knowledge emerges from lived experiences and is co-constructed through dialogue, disrupting traditional power hierarchies between researcher and research subject (58, 62). As a method, pláticas are group conversations that acknowledge the ways that communities already gather to share knowledge and build understanding. This approach was particularly salient for our research because we were able to conduct our data collection during events or meetings that groups were already organizing.

Pláticas do not only present a strategy for data collection, but also an “extension of particular ways of knowing” that challenges whose knowledge counts as legitimate within academic and policy spaces (58) (p. 102). The use of pláticas cultivates an “epistemological rupture” of dominant academic modes of inquiry and methodologies which might otherwise obscure potential insight into lived experiences (59, 63). A plática methodology allows for communal theorization of knowledge and experiences—researchers must be willing to show up authentically and answer questions being asked by contributors (58, 63, 64).

### 3.2 Conducting pláticas

Drawing from principles of community-based research, our team sought to ensure alignment with community partner organizations and residents around research goals (65). Expanding on collaborations from the Arizona Water for All network and preceding partnerships Carney had established during previous phases of community-based collaborative research conducted since 2019 in southern Arizona, potential pláticas with water-insecure communities were identified by assessing whether the conversations would be of interest and mutually beneficial for both the communities and the researchers. This first phase of research served to gather baseline data on community experiences with water insecurity while establishing the collaborative relationships and trust necessary to become part of the Arizona Water for All network, laying the groundwork for a subsequent phase of community-driven participatory planning aimed at advancing regional water security.

For each plática, there were one to two community staff from partner organizations who expressed interest in facilitating conversations. These community facilitators usually operated in a service-based capacity, such as health outreach and nonprofit work. Partnering organizations that hosted the pláticas were primarily focused on serving low-income communities. In contrast to focus groups where the interests of researchers tend to dominate the conversation, the pláticas were designed to orient discussion around topics that mattered to participants, while also circling back to the themes introduced by our university researchers (58).

University-based members of our research team corresponded with community facilitators either virtually or in person in the weeks leading up to the pláticas to co-create discussion guides and to ensure the facilitators had opportunities to ask questions and prepare for the conversations. We co-constructed a semi-structured discussion guide with these facilitators prior to convening each plática to ensure the questions were tailored to meet the unique needs of their community.

Eligibility criteria required that participants be 18 years of age or older and residents of the local community. Community facilitators helped with participant recruitment. Study objectives and researcher identities were shared during oral consent procedures with participants. The language spoken for each plática was selected by participants and community facilitators led the discussions. The majority of pláticas were facilitated in Spanish, while others included a combination of Spanish and English. Farsi was used in one plática that consisted entirely of Afghan refugees, for which the community facilitators and bilingual contributors helped to provide interpretation to the university researchers. Pláticas were convened in communal spaces frequented by the facilitators, such as community health centers, family resource centers, and a community farm. Conversations were audio recorded and hosted during existing programming, or held on weekends or evenings to accommodate participants’ schedules. Lasting between an hour to 90 minutes, participants were provided with a modest cash payment and refreshments to thank them for their time. Facilitators working outside the capacity of their organizations received a stipend as part of their collaborative role.

In total, we conducted nine pláticas with six different partner organizations across three southern Arizona counties, specifically Pima, Santa Cruz, and Cochise between February 2025 and March 2026. Each plática consisted of 7–12 participants, not including the university researchers and community facilitators, amounting to over 100 total participants. Participants were residents of southern Arizona, primarily low-income, and between the ages of 20 and 60, with the majority in their 30s and 40s. Five out of six participants were women. Almost all participants were parents with children living at home and more than half spoke Spanish as their first language and had migrated from Mexico or had grown up and continued to live on both sides of the US-Mexico border. Other countries of origin represented included Afghanistan, Peru, and Brazil.

Community-based collaborative research, like ethnographic research, is a co-constructed process wherein what people say, how it is said, and how it is received by others, or taken up and recorded via field notes is based on the quality of interactions (66). Following each plática, after participants left, the researchers debriefed with the facilitators about initial impressions and future directions. The university researchers took detailed field notes before, during, and after each plática. These were incorporated and integrated with notes shared amongst the researchers and community facilitators to cross-reference contextual and content-based interpretations of what was shared within the group.

It is worth noting that at the time of our research, reports were circulating of heightened presence of immigration enforcement, including multiple high-profile immigration raids on workplaces and other sites frequented daily by people of a migrant background in southern Arizona. As shared with the university researchers by community partners, these circumstances prevented many people from leaving their homes and participating in programming offered by these partners, and thereby may have affected outreach and recruitment of participants for the pláticas.

Data saturation was reached approximately at the sixth of nine pláticas; by this point, iterative thematic analysis revealed that no new themes, subthemes, or meaningful patterns were emerging from the data, and subsequent conversations served only to reinforce and deepen the themes already identified.

### 3.3 Analysis

Data management included storing transcripts and field notes on password protected, encrypted servers. To keep participants’ information confidential, names were not used. The university researchers were interested in determining concerns and experiences elaborated by participants; the purpose of conversations such as these is to identify priority concerns. Therefore, while a deductive coding scheme was utilized to examine instances of commonly attributed aspects of resource insecurity indicated by participants and preexisting knowledge in the literature on water insecurity, we have remained flexible to new information and updated the codes as relevant.

Audio recordings were transcribed using AI-supported transcription software in the original language (Spanish or Farsi), and the manuscript was subsequently translated into English and reviewed by Carney, Rich, and Ibarra—who collectively bring native, proficient, and intermediate Spanish-language skills—to preserve cultural meanings and linguistic nuances; translated excerpts and interpretive claims were further cross-checked against field notes from Carney, Rich, and Ibarra as well as facilitator notes to ensure fidelity to the original communicative intent.

As previously mentioned, food insecurity was not explicitly asked about or coded for, but rather appeared throughout the pláticas as a critical point warranting inclusion. Coding was conducted on the fully translated and transcribed pláticas alongside all researcher and community facilitator notes, ensuring that contextual observations and relational dynamics captured in the field were integrated into the analytic process. Carney and Rich each independently developed initial codes inductively from the data, without a priori categories, generating a provisional codebook that was refined iteratively through team discussion and constant comparison across transcripts. To ensure analytic rigor and reduce the influence of any single researcher’s interpretive lens, we engaged in independent parallel coding of a subset of transcripts, after which codes and emerging themes were compared, discrepancies discussed, and consensus reached through researcher triangulation. Coded materials were then thematically organized. The cross-checking of interpreted themes against original-language field notes served as an additional layer of interpretive validation to ensure that cultural and linguistic nuances were preserved throughout the translation and coding process.

To ensure consistency between the data and findings, translated excerpts selected for inclusion in the manuscript were verified against original-language transcripts and field notes to confirm that quoted material faithfully represented participants’ intended meanings; additionally, the thematic claims advanced in the findings were reviewed collectively by all five authors to ensure that the weight and emphasis given to each theme was proportional to its prevalence and distribution across the full dataset, rather than reflecting isolated or unrepresentative instances.

In addition to collaborative data analysis among the university-based members of our team, we have engaged collaborative data analysis procedures with community partners. Beyond the extended conversations that Carney had with facilitators in the weeks and months following each plática to discuss emergent themes, we are sharing our findings with participants through community presentations, which are also serving as spaces of iterative analysis and dialogue.

Approval for this study was obtained from the Human Subjects Office at the University of Arizona. Oral consent procedures were used with all research participants.

### 3.4 Positionality and reflexivity

The research team brought a range of positionalities and standpoints to this study that shaped both the data collection process and our interpretation of findings. We reflect on these here in the interest of transparency and methodological rigor.

The data collection team consisted of Carney (white, US-born), Rich (white, US-born), and Ibarra (Chicana, US-born), all cis-women. Carney is a tenured faculty member at the University of Arizona with professional proficiency in Spanish who has spent the past two decades engaged in collaborative, community-based research with immigrant communities, many of whose members hold liminal legal status. Her long-standing relationships with some of the participating organizations predate this study; she had been actively collaborating with them prior to its initiation. This history afforded meaningful access and trust, but also required ongoing reflexive attention to the power dynamics inherent in researcher-community relationships and the potential for prior rapport to shape participants’ responses. Ibarra is a native Spanish speaker and graduate student, whose linguistic and, where applicable, cultural proximity to participants may have facilitated openness and rapport during data collection, while also raising questions about insider/outsider positionality that the team discussed throughout the research process. Rich, also a graduate student, contributed to data collection as a relative outsider to the communities studied and remained attentive to how that position informed her interactions with participants and her interpretive lens.

Wutich and Thompson are faculty members at Arizona State University and statewide leads for the Arizona Water for All Network who were not involved in data collection but contributed to study design, analysis, and interpretation. Their distance from the field sites provided an external perspective during analysis, while also limiting their direct knowledge of the relational context in which data were gathered.

As a team, we acknowledge that our collective positionalities—shaped by gender, professional status, linguistic background, institutional affiliation, and proximity to the communities we study—influenced every stage of this research. We recognize that knowledge produced through community-engaged research is co-constructed, and that our presence in and relationships with these communities are themselves part of the methodological context. We worked to mitigate potential biases through regular team debriefs, reflexive memo-writing, and member-checking where feasible, while acknowledging the limits of those practices.

## 4 Findings

Our preliminary findings suggest that lived experiences of water insecurity among migrant, refugee, and transborder households and communities could be compounding with their experiences of food insecurity and affecting dietary intake and practices in at least three important ways: specifically, through (1) the effects of tap water avoidance on water procurement and use, (2) the labor demands of procurement and management of household water supplies, and (3) the psychosocial dimensions of water insecurity. The following section explores each of these aspects of water insecurity particularly as they surface in the everyday lives and intersect with the food practices of low-income southern Arizona residents of a migrant or transborder background. The subsequent section connects these findings to existing research on food insecurity within migrant households and communities in the global north to more extensively theorize the pathways connecting food and water insecurity among these populations.

### 4.1 Effects of tap water avoidance on water procurement and use

The majority of participants described purchasing bottled or filtered water for drinking purposes in their households, in addition to paying for tap water. They related their decisions to drinking bottled or filtered water for the reasons that they either distrusted the quality of tap water and the potential risks to health it posed and/or because of organoleptic properties of the tap water (e.g., they did not like the taste or appearance of the tap water), offering for instance, how “Agua del grifo hace la comida sabe diferente” (Tap water makes the food taste different). Taste and concerns about the health risks of drinking tap water were the primary reasons for tap water avoidance offered by all participants. In response to facilitators asking if people were more likely to buy water than use their tap water for drinking, there was a resounding affirmative response across all groups.

Participants described their distrust and dislike for the tap water in a variety of ways. Those in the group of Afghan refugees explained how they believed that “In Afghanistan, the water was natural, but here it goes through several rounds of processing, and I am not confident about it.” Another added that, “Since coming to the U.S., we also feel the water here isn’t natural, so we only use bottled water.” Several participants alluded to both distrust and taste in explaining their tap water avoidance, while also stressing the cost of water, stating for instance, “Our main concern is not trusting the safety of tap water at home, so for drinking we always buy bottled water from the market…We usually buy between four to eight packs of bottled water each week. In Afghanistan, water was free, but here the costs for water and electricity are taken from our account online.” In short, they had to pay both for tap water provided by public or private utilities that they used for bathing or laundering in addition to purchasing water separately for drinking and often cooking. Similarly, someone stated:

[The tap water] doesn’t feel safe, and I don’t know how harmful it might be for us and our family. Another worry is the source of the water—we think all the water here comes from treated sewage and wastewater. Also, the taste: when we boil tap water, the tea kettle becomes coated with deposits, which shows poor quality. Another issue is cost: buying and carrying bottled water is time-consuming and expensive.

This group showed strong support for one participant’s suggestion to engage in tap water literacy and education with refugees and migrant populations in the region, explicitly stating that “there should be an awareness program to inform people about the tap water in homes and its sources. Maybe such programs exist, but immigrants are not aware of them.”

Participants from our other pláticas described similar decision-making processes and coping strategies around water access, procurement, and use. Notably, the financial resources needed to purchase water sometimes pulled away from and reduced the household food budget.

For instance, several participants across all groups alluded to applying federal benefits intended for food—such as vouchers through the Supplemental Nutrition Assistance Program (SNAP) and the Women, Infants, and Children (WIC) program—toward acquiring bottled water.

Regarding cooking specifically, participants across all groups described avoiding tap water both for reasons of taste and health-related concerns. As one participant from Afghanistan noted, “For cooking, we use tap water, but we still don’t feel confident about it.” Some participants compared using boiled water from the tap and bottled or filtered water for cooking. As illustrated in the exchange below from participants at a plática in Santa Cruz County, they cite instances in which they or people they knew worried about developing symptoms of illness from consuming the tap water:

P1: Well, people say [it’s safe to cook with boiled water]…I’ve never seen it myself, you know? Like, some people I know do it because they say it makes things taste better, like for coffee or whatever. But there are others who prefer to just use water straight from the jug or the purifier, or they use bottled water. Well, they’ve got their pros and cons, right? Like some say, how can you use bottled water when it is so expensive? Then others say, “hey, if you boil it, it’s fine, no problem.” But honestly, we don’t really know for sure..

P2: And the thing is, we only find out when we get sick…because there are lots of people who might say, like, “oh, I’ve always cooked with tap water, never really thought twice about it, no one in my family has gotten sick,” and then there are others who tell you, “no”…Yeah, [a neighbor] told me that one time she used the [tap] water for boiling her pasta and noticed the water looked weird or the color was off, and she didn’t like it, so she stopped using it after that. So, for her it was the color, it wasn’t really that they got sick or anything.

Multiple participants expressed agreement about disliking the taste of the tap water and preferring to avoid its use as much as possible for the purposes of cooking and to substitute with bottled water. Yet bottled water consumption also raised health concerns, as participants across all sites worried about contamination from plastic bottles for human health as well as for the natural environment.

Even though most participants conveyed distrust toward the tap water, many explained that financial constraints deprived them of alternatives. One of the pláticas that consisted entirely of *promotoras* working with mixed-status households featured participants noting how many of the low-income families they visited could not afford to buy filtered water so they were drinking water directly from the tap^1^. For instance,

I mean, our clients, for starters, a lot of them, they don’t have a job. They don’t have a steady income. So usually when the bill is too high, they’re looking for help to pay a bill. They cannot afford to buy bottled water, or they don’t have the jugs [to fill at water stations]. Even if they have a jug, they don’t have a place to put it, you know, like, what do you call it? A dispenser? A dispenser, they don’t have that. One of my clients, she’s a U.S. citizen, but she lived in Mexico for the longest time, so the first time I went to her house, I noticed that she still boils her water and then she uses the dispenser because she cannot afford to buy water from elsewhere.

As described by this *promotora*, many of the households she visited lacked steady income and struggled to pay for basic utilities, leaving them unable to afford water from other sources. As a result, some resorted to boiling their water at home, which was often their only practical option. While households may have preferred to install a home filtering system, many or most were renting and did not believe it was worthwhile to invest in such an expense that could cost several hundred or thousands of dollars to install and maintain.

In addition, limitations on transportation also presented barriers to water access. The group of *promotoras* again explained how those without personal transportation or access to reliable public transportation, could not haul water bottles or jugs home: “There’s no public transportation here [in Santa Cruz County], so they can’t be carrying, like, their jug or anything.” A woman from a group in the city of Tucson recalled how when she first arrived in the U.S., “one of my problems was not having a car, and the biggest challenge for me was bringing water home. Since we had to buy bottled water, not having a car to transport it was a real burden. But over time, once I got a car, that problem was solved.” Others concurred that access to a car was absolutely essential for transporting water supplies home. To help ensure a steady domestic supply, especially when transportation may have been lacking, the *promotoras* encouraged people to carry reusable water bottles and to fill them at local businesses:

And I just let them know that, you know what, at McDonald’s you can just go in, take water. Businesses and restaurants where they can get water, so they can, like, fill their water bottles…Or if I’m taking them some other stuff, I’ll usually grab some water from the gas station, I’ll get them there to fill it. I’ll just let them know, “you know what, you can go there and get some water.”

Finally, there was some indication that water insecurity may have exacerbated local food insecurity by compromising households’ capacity for food production. Many participants cited growing fruit trees, vegetables, and herbs, and that maintaining these edible plants was complicated by conditions of water insecurity. Local drought conditions in southern Arizona increased residents’ reliance on tap water for maintaining home and community gardens, especially for those households that practiced some degree of rainwater harvesting and were coping with reduced precipitation. However, intermittent water shutoffs by utilities due to maintenance issues and delayed or missed payments, as well as instances of industrial contamination by local landfills that spurred tap water avoidance, also interfered with attempts by residents to cultivate their own sources of food.

### 4.2 Labor demands of procurement and management of household water supplies

The labor required for household water management activities emerged as a significant theme of discussion across groups. Participants recounted making weekly or biweekly trips to grocery stores and water refill stations to purchase water for drinking and also cooking. Depending on the size of one’s household, trips could be more frequent. For instance, while multiple participants reported purchasing 40-bottle cases from Walmart or a similar retailer every 2 weeks, another participant lamented having to go more often as a 40-bottle case lasted only 3 days in her household. Others hauled large jugs to water refill stations outside major retailers such as Costco, Walmart, Sprouts, Walgreens, or the gas station, as discussed in the following exchange:

F (facilitator): Where do people go to buy their water?
P1 (participant): So Walmart has it and then here yeah Nogales has one and then in Rio Rico [a nearby town], we go. It’s probably been a year since they put in a water dispenser which is great.
P2: We also have one at Walgreens. At Walgreens, there is a dispenser too.
P3: And at the gas station.
P4: I buy my water at Sprouts. I like the spring water, so I buy that at Sprouts, and I know it’s a little bit more expensive. Sometimes I get it on sale.

Participants also stayed apprised of promotions being offered on bottled water, often traveling between 30 minutes to over an hour to purchase water supplies at a reduced price from wholesale retailers. For instance, it was not unusual for those in Santa Cruz County to drive the hour (or more) to the nearest Costco to purchase water:

P1: I travel a lot to Tucson, so I stop [at Costco] and get the water.
P2: I get my water at Costco because I like the bottles, it’s a little cheaper, you get more water, like more bottles [for your money].

The labor required for procuring and transporting water included the driving times between sites of procurement and the household as well as time waiting in lines at water stations and grocery stores:

D (Deyanira): How much time does this usually take, like going to the store, waiting in line, coming back, to get water?
P1: Well, if there’s a line…
P2: Time for driving, time in the store. Getting back home.
P3: I’m convinced I need a filter now [laughter].
P2: Well, just going to the store, like, my house is… Depending on where you live. Yeah. It’s at least 15 minutes from Nogales. So it’s a half hour of just driving. And…
P1: And then if there’s a lot of people. People with several jugs.
M (Megan): Yeah, so there’s lines at the water stations sometimes?
P1: Sometimes, depending what time of day you go. After five, after everybody gets out of work [there’s a line]. Yeah. But if it’s really late at night, or if you are early, [there’s no line].

Participants explained that getting water could require a significant investment of time—often at least 30 minutes just for driving, plus waiting in lines at the store or water stations, especially during busy hours, and required access to a personal vehicle. Reflecting on how onerous of a task it was, one person, cited above, even joked that they were now convinced they needed a water filter at home. Importantly, the time and other resources required to procure water may have detracted from or competed with similar labor demands around the food and nutrition-related needs of households.

In addition to amount of time demanded by visiting water procurement sites on a regular basis—taking place anywhere from every few days to every couple of weeks—participants also commented on the physical demands of this work. Participants described carrying jugs and cases of bottled water as “physically taxing.” One woman from Santa Cruz County noted, “We buy about four to eight packs of bottled water weekly, and this is a big problem for us because it’s costly and hard to transport.” By and large, participants observed that women were the ones most often performing these tasks.

M (Megan): And is it physically difficult to do that? Like carrying the water?
P1: Yes! She was carrying it. I saw it. It is! It is. It gets heavy. Yeah.
P2: We have six three-gallon jugs. So I go like every week and a half. That is my son and myself only. We don’t drink a lot of water, but it [lasts] a week, week and a half.
M: But your son helps you get it?
P2: Well, umm, sometimes. [Laughter from the group].
M: But is it mostly women in the community who are going and buying water?
P1: Yeah that’s what I’ve seen.
P3: I’ve seen men go sometimes.
P4: I have three five-gallon jugs, but I don’t carry those. My husband carries them.
P5: We have three five-gallon [containers] and it’s $2-$3 to fill each. We have to go every week.
  F (facilitator): [To fill] all of them?
  P5: Yes, because I use it to cook, to make soups. I have to use a lot of water. To clean too, I use a lot of water. It goes really fast.

Participants described carrying large water jugs as physically straining, with many women taking on the task and relying on family help when available. Households often hauled multiple heavy containers on a weekly basis because they needed the water for drinking, cooking, and cleaning, causing supplies to run out quickly. They sometimes also limited their own water consumption and hydrated with other bottled beverages, such as soda or juice, to stretch water supplies over a longer period. Several of the women participants across groups described how they had resorted to doing things on their own, sharing statements such as, “I do everything myself,” and for those of a migrant or transborder background, contrasting their experiences in the U.S. with their countries of origin where there was more of an expectation or social obligation to help others.

In addition to the labor demands described above, there were less visible aspects of managing household water supplies that were disproportionately overseen by women. These included staying informed about the quantity of bottled or filtered water available in the household, stretching water resources for various uses, and instructing about and surveilling other household members around their water use. This included mothers for instance monitoring the length of time that their children were in the shower, or invoking other water conservation measures for household members to follow such as recycling water from the shower for the toilet or watering plants.

### 4.3 Psychosocial dimensions of water insecurity

Responding to questions regarding concerns about the affordability, accessibility, and availability of water in their communities, participants expressed worry about increasing water costs, the source of their water and the future of supply, unregulated or excessive water use by industry, increasing demands on their time to manage water supplies, negative consequences for their health, and the effects of prolonged drought.

In terms of cost, participants noted how they “were all the way out here” in primarily rural, southern Arizona and the economic implications of needing to transport water across vast distances. Someone added, “We don’t know if things are okay here right now or if in a little while there won’t be any water, because we don’t know if it’ll keep coming. Things could change. Yeah. At any moment, the energy source or whatever could run dry…” Such sentiments were echoed across participants in all the pláticas.

In Tucson, they intimated anxieties about the water demands of industry, particularly artificial intelligence, amidst plans for the development of a data center. Multiple participants suggested that shifting economic and environmental conditions could translate to more demands on their time (and labor) for procuring water in the future. Women with a migrant background and who in their countries of origin had, as one woman described, “lived through conditions of scarcity,” equally expressed their disdain or frustration for the perceived carelessness of others who maybe were less mindful about their resource use and its implications for ensuring water availability for future generations.

Regarding their health, one participant stated “I think it’s concerning because we cook and bathe with the water. Most of us don’t drink it straight from the tap, but our bodies are still getting it through our food. We absorb it,” also eliciting agreement from the group. And many participants claimed that they could not verify the source of their water, regardless of what they were told by authorities. Those living within close proximity of the US-Mexico border for instance, expressed skepticism about their water’s provenance:

P1: And the water we have, well, it comes from Mexico?
P2: Ah, I mean, where does it really come from? I wonder where the water here in Nogales, Arizona, actually comes from.
F: Honestly, we don’t really know. Well, we’ll just leave that question for someone else to figure out. The doubt’s still there.

This lack of knowledge as well as prevalence of uncertainty and doubt contributed to feelings of distrust within households and communities at large toward tap water and the water being provided by local utilities. Participants across pláticas noted that there was minimal or no effort by utilities or public health authorities to provide transparency and share knowledge with residents about water management, treatment, or quality. This perceived absence by utility providers or others responsible for managing local water supplies and overseeing public health was interpreted by participants as a form of carelessness and did not help to strengthen trust toward these institutions. For many, this lack of engagement with communities by those managing water resources compounded with residents’ past experiences of environmental contamination, such as TCE that leaked into the water supply in South Tucson during the 1980s and 1990s and had inflicted signifant harm on surrounding communities in the form of rare cancers, leukemia, other life-threatening illnesses, and congenital conditions.

It is worth underscoring how the broader political environment in southern Arizona was inducing fear and anxiety among people of undocumented or refugee status, or from mixed-status households and communities, about potential encounters with immigration enforcement. These psychosocial stressors prevented many people from leaving their homes, even to pick up groceries and other essential items. Reliance on bottled water or water from refill stations possibly proved even more precarious given these circumstances.

## 5 Discussion

Findings from our ongoing community-based collaborative research in southern Arizona suggest that water insecurity could be affecting or exacerbating food insecurity through several potential pathways. These include constraints and barriers introduced around tap water avoidance, the labor required for managing household water supplies, and negative psychosocial consequences of water insecurity.

The financial cost incurred by tap water avoidance and purchasing bottled water or that sourced from retail dispensers could be interfering with household food security, such as by infringing on household food budgets and constraining food preparation and cultivation. Migrant, transborder, and displaced populations already struggle to navigate various structural constraints that hinder consistent access to nutritious and culturally desirable foods (67). Migrants encountering new food environments often perceive the local food to be less healthy and not taste as good as foods from home (68). Service providers sometimes lack awareness of these concerns about freshness and taste when doing educational outreach about cheaper food options, such as eating canned or frozen foods, and often fail to consider culturally acceptable food when asking migrants about food needs (67, 69). Concerns about the availability of fresh food are exacerbated by higher food costs or encountering poorer quality food available at food banks (67, 70). These conditions compound with new challenges around the use of food in host countries, such as learning how to cook frozen food, use new appliances, and learn what counts as “healthy” food (67, 71). People of a migrant background may avoid shopping at large grocery stores because of unfamiliar packaging, overwhelming options to choose from, a lack of culturally available foods, language barriers when communicating with staff, and, as we had observed during the time of this research, threats of immigration enforcement being present at such sites (70, 72).

Food insecurity is often associated with lower incomes and reliance on food aid programs, and the COVID-19 pandemic intensified reliance on private food assistance programs among migrant communities in the global north (68). Despite these adaptive approaches, migrants often face difficulty navigating and keeping up with required paperwork to remain eligible for food assistance programs (70). The overarching possibility of deportation deters migrants from accessing certain public services, including food assistance (73, 74). Therefore, migrants may avoid otherwise available food or water aid programs and may enact informal responses instead. Despite informal modes of navigating instances of water scarcity, mutual aid water-sharing and self-supply practices are not a feature of equitable solutions (20, 75).

Migrants may grow their own foods, or travel further to stores with desired foods (70, 72). They may buy culturally preferred foods in bulk or early in food aid cycles, purchasing cheaper foods later or skip meals when resources are limited (70, 72). Food is usually purchased only after household and medical expenses are paid for, and may be weighed against sending remittances to family back in one’s place of origin (70). Remittance practices alongside exposure to new food cultures, impact food security, nutrition, and diets in both communities of origin and destination countries (2, 68). Water insecurity is an important but overlooked variable in households having to squeeze budgets and balance other resources for addressing food insecurity.

Our findings also highlight how labor required for managing household and community water resources could be competing with time demands for food procurement generated by food insecurity. Efforts to obtain similar foods from home can be more cost prohibitive and time consuming, requiring detailed budgeting strategies (6, 68, 72). It may also be harder to find transportation to reach stores with desired foods (70). Labor performed around water procurement and management may interfere with foodwork, or vice versa, and puts additional social pressures on women. Following a gendered division of labor around social reproduction, including foodwork, women in low-income and migrant households often oversee and manage household food resources (76). Migrant foodways are (re)produced by women who are often charged with feeding and caring for household members and families (77). Consequently, women frequently endure additional health risks after migrating when conditions of food insecurity complicate their ability to perform foodwork (6, 73, 78). Women are usually the first in the household to adjust what and how much they eat and to alleviate the impact on children when food insecurity looms—they disproportionately shoulder the burden of food insecurity and are the most attuned and sensitive to resource disruptions at the household level (6, 70). Similarly, women are particularly impacted by water shortages, as responsibility for water procurement and provisioning is often assigned to them (38, 79–81). In the US Southwest where water scarcity is especially prevalent, girls are familiarized with domestic water-labor demands from an early age (81). In dedicating more time to the provisioning of water, women may also have less time to cook high quality foods, possibly leading to more consumption of poorer quality foods with nutritional deficits (38). When water resources are unreliable or scarce, more time is required to perform the labor of water procurement and provisioning, interfering with or detracting from the labor and other resources available to manage food security.

The negative psychosocial consequences of water insecurity could be compounding with psychosocial and other extant health vulnerabilities associated with household food insecurity. Importantly, food insecurity is associated with poor nutrition and negative health outcomes in displaced populations who face limited access to health and social services due to precarious legal status (83). Despite common assumptions, health status declines with migration (84). This may be partially explained by the threat of food and water insecurity that persists even after migrating. Migrants facing food insecurity are also at higher risk for psychosocial stress, amplified for instance by worries about eating in accordance with religious or cultural needs (72, 85). Women in particular, situated as they are on the “frontlines” of food insecurity, are disproportionately affected by the emotional and psychological dimensions of resource scarcity. Our findings suggest that everyday anxieties or worries about water insecurity could be compounding with the psychological and emotional toll of food insecurity in migratory or transborder contexts, with particularly pronounced effects for women.

**FIGURE 1:**
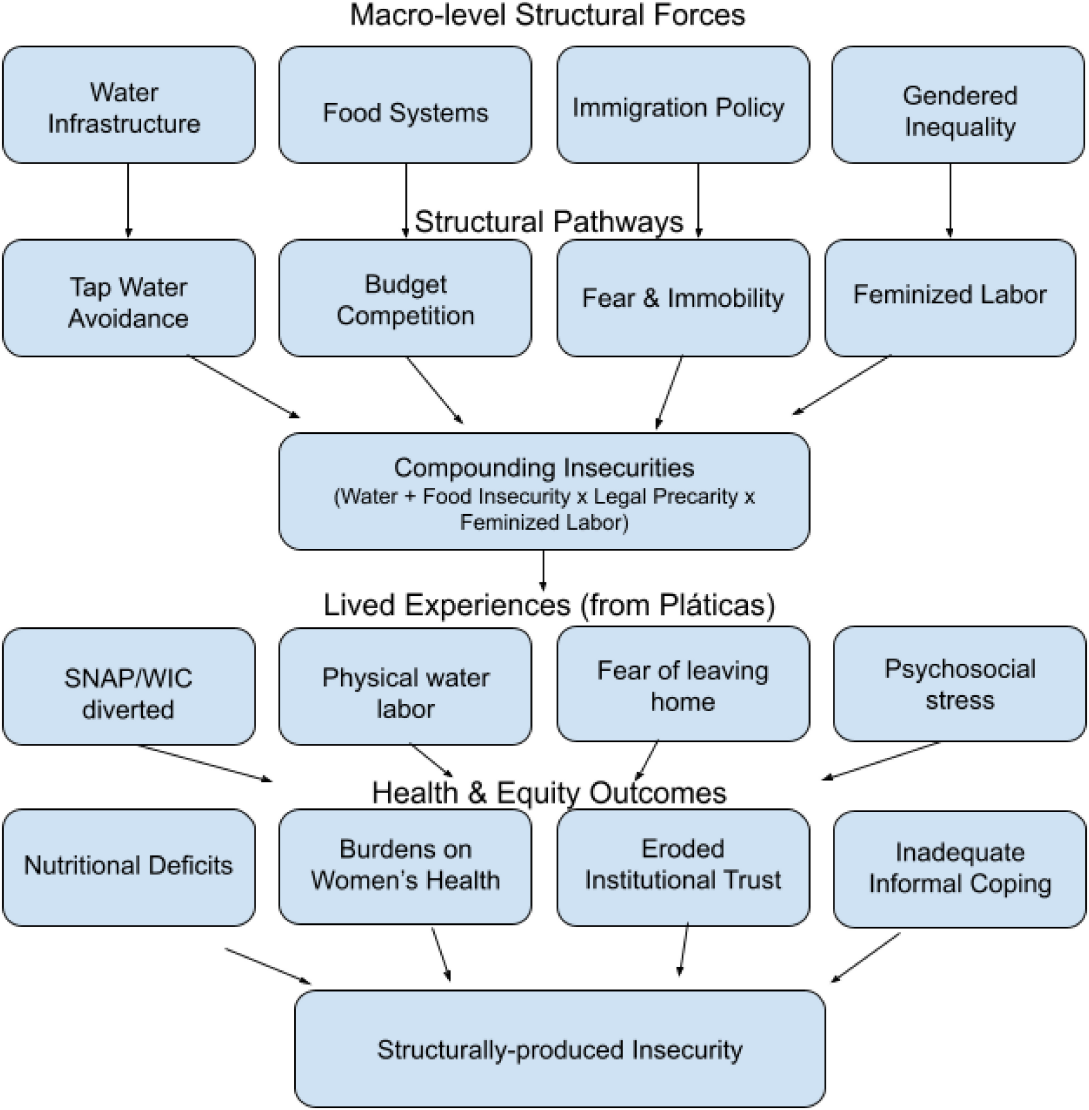
Water and food insecurity: from lived experience to structural forces.

Our findings must be contextualized within the larger structural and macro-level dynamics that shape the realm of lived experience, including but not limited to water infrastructure, food systems, immigration policy, and gendered inequality (see Figure 1). Public water systems are failing to build trust within these communities, not only through lack of communication but also deeper histories of environmental racism. In this context, distrust emerges as a rational response to infrastructural neglect rather than indicating a knowledge deficit. The commodification of water pushes households to pay twice—once for tap water they don’t always use and again for bottled or filtered water, creating competition with food budgets. The inequities in water infrastructure observed across these communities reflects a longer pattern of disvestment in the US-Mexico border region. The threat of immigration enforcement functions as a structural barrier to food and water resources, compounding with existing economic constraints. Meanwhile, formal safety nets are structurally inaccessible to many mixed-status households, leading to increased reliance on informal and inadequate coping strategies. Women are especially structurally vulnerable to the health inequities produced by these compounding insecurities, situated as they are on the frontlines of managing household food and water resources. In summary, water and food insecurity in migrant and transborder communities is best understood not as a discrete household problem but as a structural condition that demands upstream interventions.

## 6 Conclusion

Emerging evidence from community-based collaborative research conducted in southern Arizona highlights lived experiences and perspectives of water insecurity among migrant, transborder, and displaced populations. Our research responds to existing calls for more scholarly and policy attention to the ways that migrant populations experience and navigate water insecurity in high-income countries, and offers new insights into the linked pathways between water and food insecurity in such contexts. Using a plática methodology, participants discussed their water-based decision-making which often involved the avoidance of tap water; the amount of time and other resources that were mobilized in managing household water supplies; and their health-related concerns as well as concerns around the future of water in this arid, drought-stricken setting.

Coalitional research requires reflexivity regarding the power dynamics shaping research projects. In continuing to think about the use of pláticas, it is critical to remain attentive to ethical considerations such as place, timing, and the way data is collected to ensure pláticas maintain rigor and uphold an equity-imbued practice, and we acknowledge that pláticas may not always represent the best approach (60).

In this regard, we should underscore some of the limitations of a pláticas methodology. The specific context of Arizona’s borderlands, while critical for advancing knowledge of compounding instances of water and food insecurity in migratory contexts, is nonetheless geographically and contextually unique. Hence, the perspectives of participants offered through the pláticas may not be generalizable for all migrant, transborder, or displaced populations experiencing water insecurity. At the same time, a pláticas methodology prioritizes depth, trust, and cultural resonance over breadth, and findings are not meant to be statistically generalizable. In addition, researcher positionality, shaped by identity, lived experiences, and community ties may affect what is shared (or not), and how it is interpreted. Translation and interpretation present additional layers of complexity that may risk flattening or distorting culturally embedded knowledge. Rooted in relational, community-centered inquiry, a pláticas methodology implies inherent limitations that are inseparable from its interpretive commitments.

While our study lacked an explicit focus on food insecurity, our findings point to some of the interactions among and compounding effects of water and food insecurity as they shape the health and livelihoods of migrant, transborder, and displaced communities. These interactions warrant further attention by researchers, practitioners, and policymakers. Future research exploring the connection between migration or human mobility and dietary transformations and health implications in other settings of the global north should also consider migrant populations’ encounters with and responses to water insecurity. Meanwhile, practitioners and policymakers seeking to address food insecurity and health among migrant and transborder populations must be attentive to conditions of water insecurity and other resource-related challenges that will very likely continue to intensify in the context of global climate change.

## Data Availability

The datasets presented in this article are not readily available because IRB protocol does not permit us making these data widely available. Requests to access the datasets should be directed to Megan Carney, mcarney@arizona.edu.

## Footnotes

1 The Arizona Department of Health Services describes a *promotora* as “a community health worker who’s a trusted member of the community and serves as a link between people and services to promote the overall health of family, friends and neighbors.”
